# Controlling wave-vector of propagating surface plasmon polaritons on single-crystalline gold nanoplates

**DOI:** 10.1038/srep13424

**Published:** 2015-08-25

**Authors:** Si Luo, Hangbo Yang, Yuanqing Yang, Ding Zhao, Xingxing Chen, Min Qiu, Qiang Li

**Affiliations:** 1 State Key Laboratory of Modern Optical Instrumentation, College of Optical Science and Engineering, Zhejiang University, Hangzhou 310027, China; 2 School of Information and Communication Technology, KTH Royal Institute of Technology, Electrum 229, 16440 Kista, Sweden

## Abstract

Surface plasmon polaritons (SPPs) propagating at metal nanostructures play an important role in breaking the diffraction limit. Chemically synthesized single-crystalline metal nanoplates with atomically flat surfaces provide favorable features compared with traditional polycrystalline metal films. The excitation and propagation of leaky SPPs on micrometer sized (10–20 μm) and thin (30 nm) gold nanoplates are investigated utilizing leakage radiation microscopy. By varying polarization and excitation positions of incident light on apexes of nanoplates, wave-vector (including propagation constant and propagation direction) distributions of leaky SPPs in Fourier planes can be controlled, indicating tunable SPP propagation. These results hold promise for potential development of chemically synthesized single-crystalline metal nanoplates as plasmonic platforms in future applications.

In recent years, considerable interests have been taken in the development of metal nanostructures that support surface plasmon polaritons (SPPs)^1^,^2^,^3^,^4^. Due to their strong localization beyond the diffraction limit and consequent field-enhancement properties, the SPPs are employed in extensive applications ranging from biosensing^5^,^6^,^7^,^8^,^9^, high resolution imaging^10^,^11^, to plasmonic circuitry^12^,^13^,^14^,^15^,^16^. Generally, the aforementioned SPPs fall into two categories: propagating SPPs or localized SPPs. On one hand, for the propagating SPPs, different waveguide nanostructures have been fabricated on vapor-deposited or sputtered polycrystalline metal films^17^,^18^,^19^. However, the polycrystalline character of these metal films introduces intrinsic surface roughness which causes additional scattering and thereby increases propagation loss of SPPs^20^,^21^. On the other hand, chemically synthesized metal nanoparticles, such as nanorods^22^, nanocubes^23^, nanostars^24^ or nanoprisms with a size of a few hundred nm^25^,^26^,^27^, which provide high surface quality with single-crystalline feature, have been widely used for localized SPPs. Large (>100 μm^2^) single-crystalline metal nanoplates have also been synthesized by wet chemical methods. For example, Au nanoplates are demonstrated as ultra-smooth basis for nanoantennas to study the localized SPPs^28^. So far, few researches have been put forward on propagating SPPs on large single-crystalline metal nanoplates^29^,^30^,^31^. Ref. [29] has shown the polarization control of propagating SPPs on Au nanoplates with an average size of 5 ± 2 μm and an average thickness of 107 ± 30 nm by real image detection; however, the propagation direction and the propagation constant of SPPs cannot be examined in the real image detection. To overcome this drawback, a method of milling slits by focused ion beam (FIB) on nanoplates has been adopted^30^,^31^, which can study the propagation direction and the propagation length of SPPs on nanoplates. Ref. [30] has studied the propagation direction of Ag nanoplates with a thickness of 100–200 nm by placing a nanobelt or milling a nanogap on surfaces. Ref. [31] has measured the propagation length of SPPs on Ag nanoplates through detecting the scattered light of silts milled by FIB. However, the milling method cannot measure the effective refractive index. Besides, it damages the integrity of nanoplates to some extent. Noteworthy, leakage radiation microscopy (LRM), which has been used to characterize the propagation of leaky SPPs in plasmonic nanowires^32^,^33^, detects leaky radiation of propagating SPP modes into substrates by imaging of the back focal plane (BFP). The LRM provides a damage-free characterization method and can be utilized to further study the wave-vector (including both propagation constant and propagation direction) of SPPs in large gold nanoplates.

In this paper, controlling the propagating leaky SPPs on chemically synthesized single-crystalline gold nanoplates is characterized by the LRM. Firstly, both synthesis of gold nanoplates and LRM setup for characterization are presented. These nanoplates possess large sizes of tens of micrometers as well as well-defined crystal orientations with atomically flat surfaces. Secondly, for triangular nanoplates, wave-vector distributions of two kinds of leaky plasmon modes are analyzed in far-field patterns on BFPs. The excitation position and polarization of incident light on the apex of nanoplates are manipulated to control the propagating characteristics of leaky SPPs visualized in both the real image plane and the Fourier plane. Thirdly, the propagation properties of leaky modes in different edges for hexagonal nanoplates are studied. Finally, the conclusion is given.

## Methods

### Synthesis of gold nanoplates

**Figure 1:**
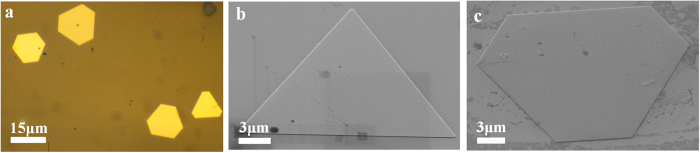
(**a**) Typical optical microscope image of gold nanoplates. (**b**) and (**c**) are SEM images of triangular and hexagonal nanoplate, respectively. The images are taken with 54° tilting angles for the sample stage.

The growth of gold nanoplates used in experiments is achieved following the chemical synthesis procedure described in previous reports^28^,^34^. The gold nanoplates are washed with anhydrous ethanol several times and dispersed on an indium tin oxide coated cover glass substrate (n = 1.52). Figure 1 shows a typical optical microscopy image and scanning electron microscope (SEM) images of nanoplates including shapes of both equilateral triangle and hexagon. The area of nanoplates can reach about 300 μm^2^ and nanoplates with an edge length of tens of micrometres are chosen in experiments to investigate the propagating SPPs. The thickness of nanoplates used in experiments is around 30 nm, thus supporting leaky radiation of propagating SPPs into the substrate.

### Optical setup

**Figure 2:**
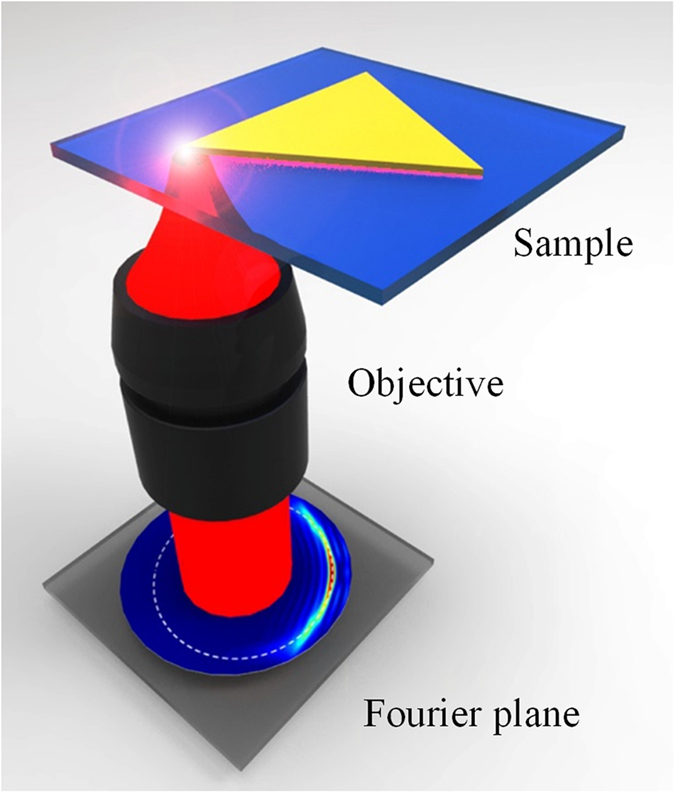
Sketch of optical excitation of SPPs at 980 nm in a triangular gold nanoplate. The wave-vector distributions of the leaky radiation are recorded in the Fourier plane.

The sketch of the experimental setup is shown in Fig. 2. The sample is placed on a homebuilt sample stage with a three-axis adjustment holder controlling its position. Linearly polarized incident light of 980 nm from the substrate is focused with a high numerical number (NA) objective (Nikon, 100×, NA = 1.49 immersion, inverted) to excite the SPPs on the gold nanoplates. The objective collects leaky radiation of the propagating SPPs. The reflection of the incident light on the sample can be filtered by an adjustable aperture installed in the image plane of the objective. The propagation properties of SPPs in the real image plane are visualized on an infrared CCD. The corresponding wave-vector distributions are recorded in the Fourier plane of the objective.

## Results and Discussions.

**Figure 3:**
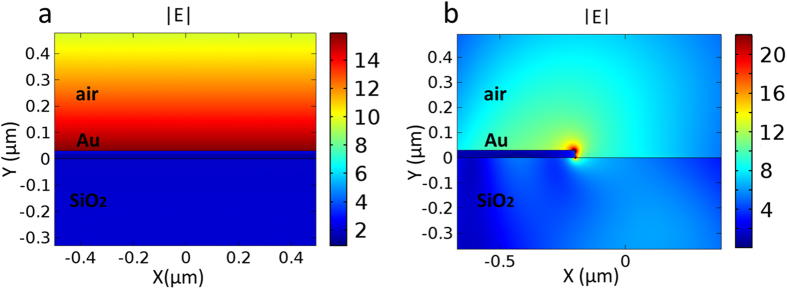
Simulated electric field distributions of two kinds of leaky SiO_2_-Au-air SPP modes: (**a**) planar mode and (**b**) corner mode.

Leaky mode analysis on synthesized nanoplates. There are two kinds of leaky modes that can be detected by the LRM at 980 nm wavelength. The first kind is SiO_2_-Au-air SPP planar mode (termed as “planar mode”) propagating mainly on the Au nanoplate surface. The other kind is SiO_2_-Au-air SPP corner mode (termed as “corner mode”) propagating along the nanoplate edges. The field distributions of these two modes (calculated from FDTD) are shown in Fig. 3a,b, respectively. The effective refractive indexes of the planar mode and corner mode are calculated to be 1.0168 and 0.971, respectively. The leaky SiO_2_-Au (30 nm)-air SPP planar mode can be regarded as Au-air SPP planar mode slightly affected by the substrate. Therefore, its simulated effective refractive index (1.0168) is close to that of Au-air SPP planar mode (1.0127).

### Controlling SPP corner modes on triangular nanoplates by excitation position manipulation

**Figure 4:**
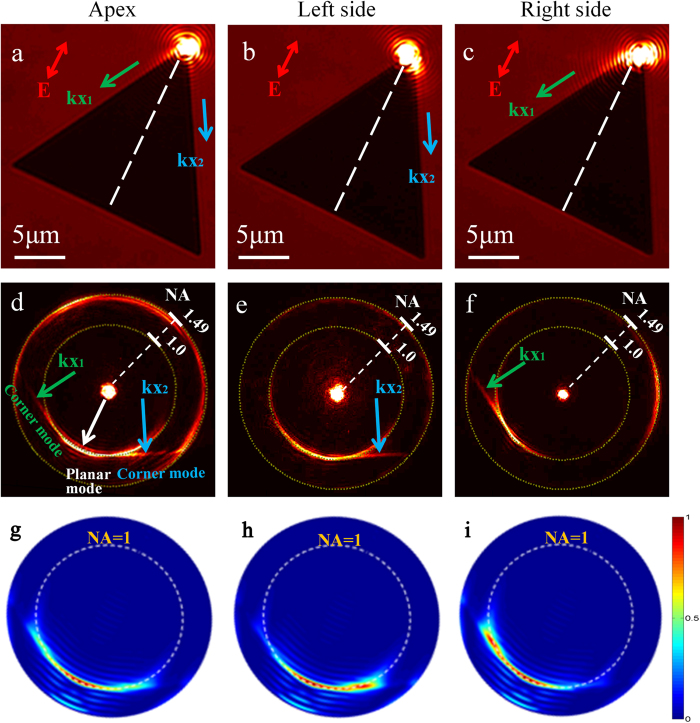
Experimental real (**a–c**) and Fourier (**d–f**) images for different excitation positions. (**g,h**) are FDTD simulation results of corresponding Fourier images.

In our experiments, the light is incident from the substrate side and focused onto the apex of the triangular nanoplate. The polarization of the incident laser is aligned in parallel with the bisector of the apex angle, shown as the red arrow in Fig. 4a. The scattering of SPP corner mode (kx_1_ and kx_2_) in two directions along both edges from the apex are observable in the bright field of the real image plane (Fig. 4a).

The corresponding Fourier image is depicted in Fig. 4d, manifesting the wave-vector distributions of the propagating SPP leaky modes. Most of the incident light is filtered out in the Fourier plane. The radial coordinate on the Fourier image indicates effective refractive index (n_eff_) of leaky SPPs. The yellow dashed outer circle on the Fourier image represents n_eff_ = 1.49, which is the NA of the oil immersion objective. The inner circle on the Fourier image demonstrates n_eff_ = 1.0, defined by the critical angle of the air-glass interface. Two bright straight lines indicate the wave-vector distributions of the corner modes on the triangular nanoplate in the Fourier plane and the measured effective refractive indexes are 0.98. More importantly, the directions of the two straight lines in the Fourier plane are perpendicular to the edges of the gold nanoplate, indicating the two propagation directions along the edges of the nanoplate. Besides, a bright circular arc is also shown on the Fourier image with the effective refractive index quite near unity. It results from the excited SPP planar mode propagating in different directions over the whole nanoplate surface. The extra fringes shown in the Fourier plane are associated with interference between reflected SPPs from the edges and propagating SPPs. The number of the periodic fringes thus depends on the size of gold nanoplates^33^.

In order to further unveil the propagation properties of the corner modes on the edges of the nanoplate, the focusing position of the incident light is moved to the left or right side about several hundred nanometres away from the apex. As is shown in Fig. 4b, when the incident light is focused on the left, the SPPs propagate mostly along the right edge. When the light spot is moved to the right side, the SPPs propagating along the left edge can be observed, as is shown in Fig. 4c. The corresponding BFP patterns are indicated in Fig. 4e,f, each obviously showing one bright line consistent with the propagation direction of corner mode in the real image plane.

Additionally, the simulation results based on the finite-difference time-domain (FDTD) method for different focusing positions of incident light are presented in Fig. 4g–i. When the light spot is on the apex, both bright arcs and two straight lines are obtained (Fig. 4g). When the light spot is moved to the left side of the apex, only one straight line is shown on the Fourier image (Fig. 4h), indicating that corner mode at the right side is much stronger than that at the other side. The slight difference between simulated and experimental results can be attributed to uncertainty of the spot position and inevitable chemical impurities on the synthesized nanoplates. The control of the focusing position provides a novel avenue of manipulating propagation directions of leaky SPPs on the nanoplate.

### Controlling SPP corner modes on triangular nanoplates by polarization manipulation

**Figure 5:**
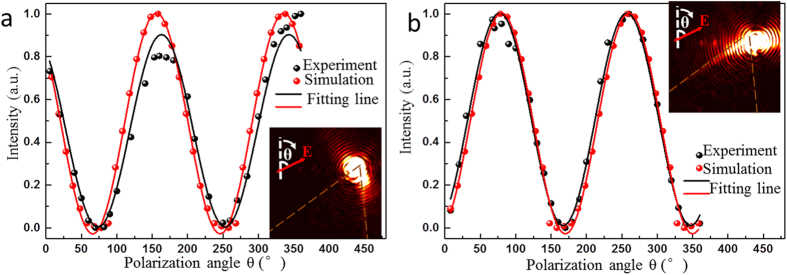
Normalized intensity of leaky radiation of SPP corner modes extracted from Fourier images as a function of laser polarization. The light is focused on the left side (**a**) and right side (**b**), which are indicated in the insets. The black and red dots denote the experimental and simulated results, respectively. The lines denote fitting using sine functions.

In this part, another method of manipulating propagation directions of SPP corner modes on nanoplates by polarization control of the focused light is investigated. The light is focused at the same positions as those in Fig. 4b,c. The intensity of the bright straight line on the corresponding Fourier images is recorded every 10° or 20° of the polarization angle in a period of 360°. Figure 5 shows the normalized intensity of the leaky radiation of SPP corner modes propagating along the edges as a function of the laser polarization together with sine fitting functions. In Fig. 5a, when the incident laser is focused on the left side near the apex, the maximum leaky radiation intensity corresponds to a polarization angle of 163° in the experiment, which is approximately parallel to the direction of SPPs propagating along the right edge. The minimum leaky radiation intensity on the BFP occurs at the polarization angle of about 73°, nearly parallel to the left edge. A similar situation occurs when the light is incident at the right side near the apex, as is illustrated in Fig. 5b. The maximum and minimum intensity happens when the polarization angle is 78° and 168°, respectively.

The experiment results are also verified by the FDTD simulation results, which are provided in Fig. 5. The simulation results agree well with the experimental results except that there are slight derivations in the polarization angle corresponding to peak or valley intensity. The manipulation of light polarization to modulate SPP intensity on the nanoplate is similar to that on the nanowire^35^, however, the peak intensity does not strictly correspond to the polarization parallel to the edges for nanoplates, which can be attributed to the more complex geometric structure compared with the one-dimension nanowire.

### Controlling SPP corner modes on hexagonal nanoplates

**Figure 6:**
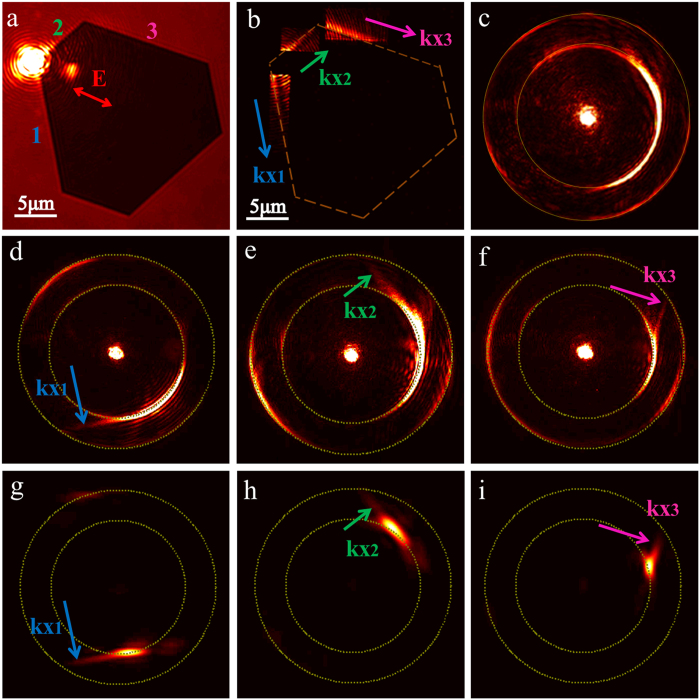
Experimentally recorded Fourier images of hexagonal gold nanoplates. (**a**) The bright field image in the real image plane of the hexagonal gold nanoplate with labelled edges (1, 2 and 3). The red arrow represents the polarization of incident light. (**b**) The dark field images in the real image plane of SPP corner modes propagating along three labelled edges. (**c**) The Fourier image of propagating SPP modes in all different directions under the polarization state in (**a**). By adjusting the polarization of incident light, the Fourier images correspond to intensity peaks of the three propagation directions (kx_1_, kx_2_ and kx_3_) are shown in (**d**) to (**f**). By extracting the propagating leaky SPP corner mode in one direction (kx_1_, kx_2_ or kx_3_) in the real image plane, the corresponding Fourier images are shown in (**g**) to (**i**).

The propagating properties of leaky SPP corner modes on hexagonal nanoplates are further studied. As is shown in Fig. 6a, when the incident laser is focused on the apex of a hexagonal nanoplate, the excited SPPs propagate along the edges as well as the whole surface of the nanoplate. Figure 6b depicts three individual dark field images of SPP corner modes propagating along the labelled edges in the real image plane. However, due to superposition and interference of propagating SPPs in all different directions, it is too ambiguous and complicated to identify the wave-vectors of the SPP modes propagating along the edges, as is shown in Fig. 6c.

In order to clearly identify the three wave-vectors (kx_1_, kx_2_ and kx_3_) of SPP corner modes along the three individual edges (1, 2 and 3), two methods are adopted in the experiment. For the first method, the polarization of incident light is adjusted to maximize the excitation of SPP corner modes along one specific direction. This method exhibits the advantage of enhanced intensity of leaky SPP mode along one specific edge and thus can be used to control SPP propagation on hexagonal nanoplates. As are shown in Fig. 6d–f, three straight lines correspond to leaky SPP corner modes propagating along three individual edges can be clearly observed. For the second method, a square adjustable aperture is inserted to extract the leaky SPP corner modes propagating along the three individual edges for Fourier imaging; therefore, enhanced signal-noise-ratio for leaky SPP corner modes propagating along one direction can be realized. The corresponding Fourier images are provided in Fig. 6g–i. The obtained wave-vectors of leaky SPP corner modes are the same as those shown in Fig. 6d–f. The obtained effective refractive indexes of leaky SPP modes on the hexagonal nanoplate are close to those on the triangle nanoplate. Hexagonal nanoplates provide more degrees of freedom for manipulating the wave-vector of leaky SPPs on metal/air interfaces compared with triangular nanoplates.

To conclude, by means of leakage radiation microscopy, the propagating properties of leaky SPPs on single-crystalline, micrometre-sized gold nanoplates have been studied. The wave-vectors of two kinds of modes (planar mode and corner mode) are analyzed. Both the propagation constant and propagation direction of leaky SPP modes on triangular and hexagonal nanoplates are obtained in the Fourier planes. The effective refractive indexes for the leaky SPP corner modes on triangular and hexagonal nanoplates are measured to be around 0.98. For triangular nanoplates, by manipulating the focusing position and polarization of incident light, the propagation directions and intensity of corner modes can be controlled between the two edges. When the focusing position of incident light is at the left (right) side of the apex, the leaky SPP corner modes propagating along the right (left) edge can be effectively excited. When the polarization of the incident light is adjusted, the intensity of the leaky SPP corner modes follows a sine function with respect to polarization angles. For hexagonal nanoplates, three wave-vectors of SPP corner modes are clearly demonstrated, thereby providing more degrees of freedom for manipulating the wave-vector of leaky SPPs in the SiO_2_-Au-air structure. The results demonstrated here are beneficial for applying such chemically synthesized single-crystalline metal nanostructures in future applications.

## Additional Information

**How to cite this article**: Luo, S. *et al*. Controlling wave-vector of propagating surface plasmon polaritons on single-crystalline gold nanoplates. *Sci. Rep*. **5**, 13424; doi: 10.1038/srep13424 (2015).
